# Distinct Signatures of Genomic Copy Number Variants Define Subgroups of Merkel Cell Carcinoma Tumors

**DOI:** 10.3390/cancers13051134

**Published:** 2021-03-06

**Authors:** Natasha T. Hill, David Kim, Klaus J. Busam, Emily Y. Chu, Clayton Green, Isaac Brownell

**Affiliations:** 1 Dermatology Branch, National Cancer Institute, NIH, Bethesda, MD 20892, USA; natasha.hill@nih.gov; 2 Marshfield Center, Marshfield, WI 02050, USA; kim.seung@marshfieldclinic.org (D.K.); clayton_green@urmc.rochester.edu (C.G.); 3 Department of Pathology, Memorial Sloan Kettering Cancer Center, New York, NY 10065, USA; busamk@MSKCC.ORG; 4 Deprtment of Dermatology, Perelman School of Medicine at the University of Pennsylvania, Philadelphia, PA 19104, USA; Emily.Chu@uphs.upenn.edu

**Keywords:** Merkel cell carcinoma, virus positive Merkel cell carcinoma, virus negative Merkel cell carcinoma and copy number variant

## Abstract

**Simple Summary:**

Cancer results from genetic changes in cells. These changes are often mutations that alter the DNA sequence of critical genes. However, duplications and deletions in cancer-related genes can also contribute to malignant transformation. In this study we use Nanostring technology to assess DNA copy number changes in samples of Merkel cell carcinoma (MCC), a rare and aggressive neuroendocrine skin tumor. We were able to identify recurrent amplifications and deletions in cancer-related genes. We also found that MCC tumors grouped into three distinct copy number variant profiles. The first group consisted of tumors with multiple deletions. The second group contained tumors with low levels of genomic structural alterations. The last group comprised tumors containing multiple amplifications. Our study suggests that most MCC tumors are associated with deletions in cancer-related genes or are lacking in copy number changes, whereas a small percentage of tumors are associated with genomic amplifications.

**Abstract:**

Merkel cell carcinoma (MCC) is a rare, aggressive neuroendocrine skin cancer. Most MCC tumors contain integrated Merkel cell polyomavirus DNA (virus-positive MCC, VP-MCC) and carry a low somatic mutation burden whereas virus-negative MCC (VN-MCC) possess numerous ultraviolet-signature mutations. In contrast to viral oncogenes and sequence mutations, little is known about genomic structural variants in MCC. To identify copy number variants in commonly altered genes, we analyzed genomic DNA from 31 tumor samples using the Nanostring nCounter copy number cancer panel. Unsupervised clustering revealed three tumor groups with distinct genomic structural variant signatures. The first cluster was characterized by multiple recurrent deletions in genes such as *RB1* and *WT1*. The second cluster contained eight VP-MCC and displayed very few structural variations. The final cluster contained one VP-MCC and four VN-MCC with predominantly genomic amplifications in genes like *MDM4*, *SKP2*, and *KIT* and deletions in *TP53*. Overall, VN-MCC contained more structure variation than VP-MCC but did not cluster separately from VP-MCC. The observation that most MCC tumors demonstrate a deletion-dominated structural group signature, independent of virus status, suggests a shared pathophysiology among most VP-MCC and VN-MCC tumors.

## 1. Introduction

Merkel cell carcinoma (MCC) is a rare neuroendocrine skin cancer associated with advanced age, UV-damage, and immunosuppression [1,2,3]. MCC is an aggressive cancer, with a lethality rate of over one-third, and thus is more deadly than malignant melanoma [1,3,4,5]. The incidence of MCC has increased in the past several decades in part due to improved diagnostic tools, increased clinical awareness, an aging population, and increased sun exposed skin [2,3]. In the United States, approximately 50–80% of MCC tumors are Merkel cell polyomavirus-positive (VP-MCC), with clonal integration of viral DNA into the host genome [6,7,8,9,10,11,12]. VP-MCC tumors carry a low somatic mutation burden, suggesting that tumorigenesis is driven by viral T antigen oncogenes [11,13,14,15,16,17,18,19]. The remaining 20–50% of MCC tumors are polyomavirus-negative (VN-MCC) and possess numerous ultraviolet signature mutations in genes such as *p53* and *RB1* [6,8,9,10,11,12,13,14,15,16]. Although a number of molecular and cytogenetic alterations have been reported for MCC, no unique signatures have been identified [11,13,14,15,16,20,21].

Genomic instability can initiate cancers, contribute to disease progression and impact patient response to treatment [22,23,24]. Several factors promote genomic instability leading to genomic structural variants in the form of amplifications or deletions, such as telomere damage, epigenetic modifications and DNA damage [22,23]. Here we use Nanostring’s nCounter copy number variant (CNV) analysis to identify commonly amplified or deleted cancer-related genes in MCC. Unsupervised clustering identified three tumor groups with distinct genomic structural variant signatures. On average VP-MCC tumors had fewer copy number changes than VN-MCC. Furthermore, the cluster of tumors characterized by very few structural variants were all VP-MCC. In contrast, the tumors with numerous copy number variants clustered independently of virus status, suggesting a shared genomic instability among VN-MCC and a subset of VP-MCC.

## 2. Results

### 2.1. Three Genomic Structural Variant Signatures Identified in MCC Tumors

**Figure 1:**
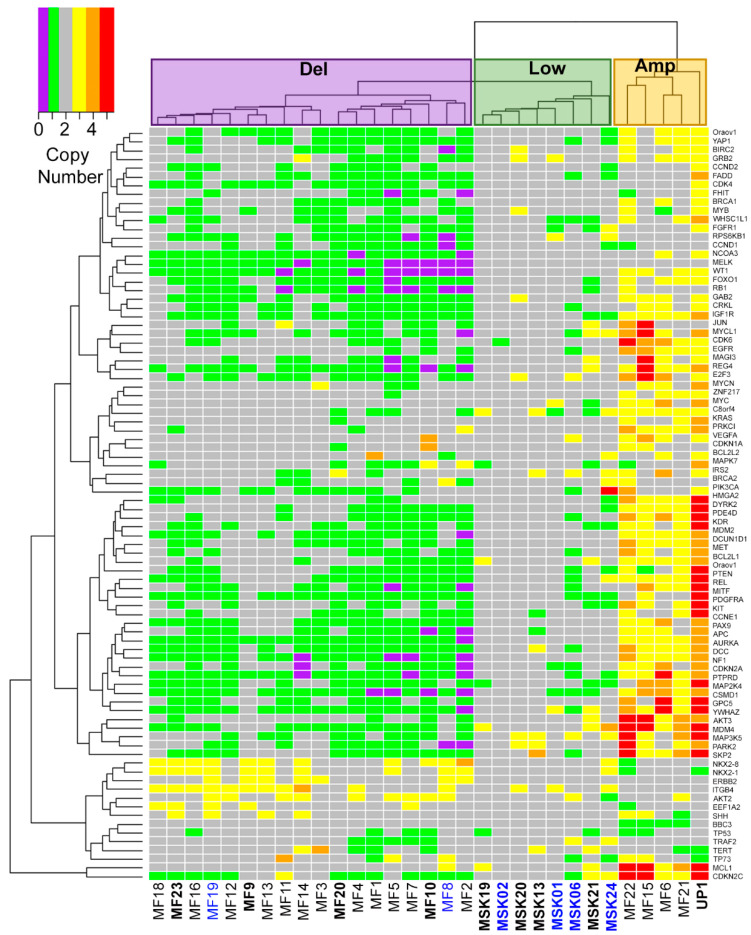
Three genomic structural variant signatures detected in MCC tumors by NanoString nCounter. Tumor DNA from 31 patients with MCC from Memorial Sloan Kettering (MSK), Marshfield Clinic (MF), and the University of Pennsylvania (UP) were subjected to Nanostring nCounter CNV analysis. CNV alterations for 86 gene loci commonly altered in cancer were ascertained and plotted as a heatmap. Three cluster groups denoted as Del (deletion) for group 1, Low for group 2, and Amp (amplification) for group 3. Bold indicates virus positive MCC (VP-MCC) tumors. Blue indicates metastatic tumor.

Despite the importance of genomic integrity in cancer, little is known about the genomic structural variants that lead to MCC. Therefore, we sought to identify commonly amplified or deleted cancer genes in MCC. We obtained 31 MCC tumors from Memorial Sloan Kettering (MSK), Marshfield Clinic (MF), and the University of Pennsylvania (UP) (Table 1). The patients ranged from 53 to 100 years of age. Most of the tumors analyzed were obtained from primary tumor lesions on sun exposed skin (Table 1). Genomic DNA from tumor samples and control tissues was analyzed using Nanostring Technologies’ copy number variant cancer panel assay. Fresh-frozen tumors from MSK were normalized to fresh-frozen adjacent tissue samples, whereas FFPE tumors were normalized to FFPE normal spleen samples. As depicted in Figure 1, unsupervised clustering identified three distinct structural variant groups. Tumors clustered in group 1 (Del) displayed numerous recurrent deletions in a number of genes, including genes involved in cycle regulation such as *RB1* (Figure 1 and Table S1). Tumors in group 2 (Low) showed very few genomic structural variations. In the third group (Amp), tumors carried very few deletions but contained numerous recurrent amplifications in several genes, including *MDM4*, *AKT3*, *BCL2L1* and *MYCL1* (Figure 1 and Table S1). In this cohort of 31 MCC tumors, most of the tumors (18, 58%) have the structural group 1 Del signature dominated by deletions in cancer related genes. Group 2 Low with few changes accounted for 8 (26%) tumors, whereas only 5 (16%) tumors had the amplification-heavy group 3 Amp signature.

### 2.2. MCC Structural Variant Signatures Are Characterized by Deletions, Absence of Copy Changes, or Amplifications

**Figure 2:**
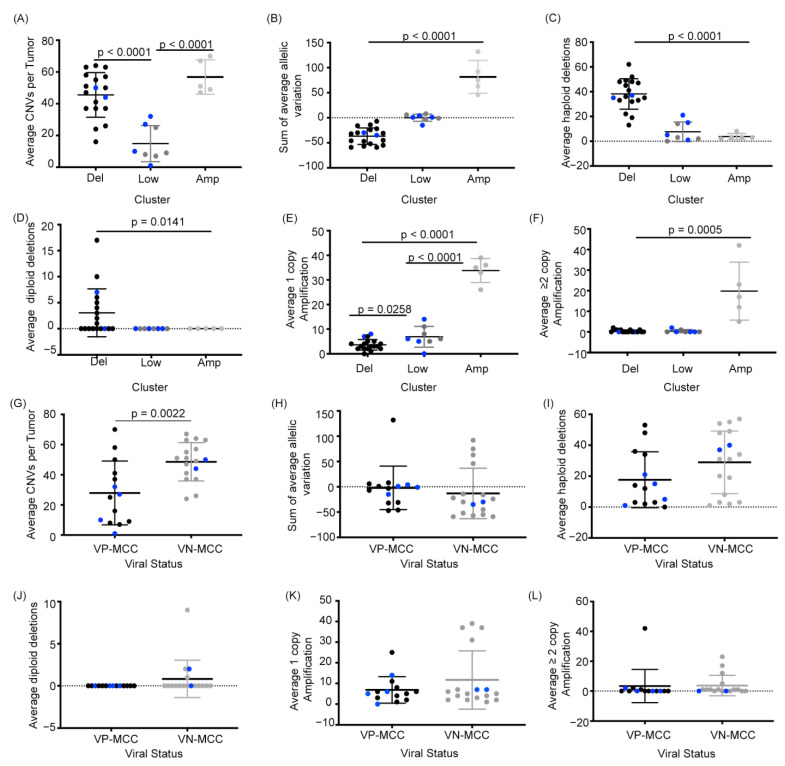
Structural variant clusters show distinct levels and types of CNVs whereas VN-MCC show more structural variation than VP-MCC. Comparison of clusters for the (**A**) average number of CNVs per tumor (one-way ANOVA), (**B**) average sum of allelic variations (−1 for each haploid deletion, +1 for each amplification, Kruskal-Wallis test), (**C**) average haploid deletions (Kruskal-Wallis test), (**D**) average diploid deletions (Kruskal-Wallis test), (**E**) average single-copy amplification (one-way ANOVA), and (**F**) average two or greater copy amplifications (Kruskal-Wallis test). Comparison between VP-MCC and VN-MCC for the (**G**) average number of CNVs per tumor (unpaired *T*-test), (**H**) average s um of allelic variations (Mann-Whitney test), (**I**) average haploid deletions (unpaired *T*-test), (**J**) average diploid deletions (Mann-Whitney test), (**K**) average single-copy amplification (Mann-Whitney test), and (**L**) average two or greater copy amplifications (Mann-Whitney test). Uncolored dots are primary tumor samples, blue dots indicate metastatic tumor samples.

To characterize the differences between MCC structural variant groups we compared the total number of copy number variations per tumor for each cluster. Tumors in both the Del and Amp groups had significantly more CNVs per tumor than tumors in the Low group (*p* < 0.0001, Figure 2A). We then compared the average sum of the allelic variation relative to diploid (−1 for each allelic deletion, +1 for each amplification, total of 86 genes) for the tumors in each cluster. As seen in Figure 2B, the Del group tumors had the lowest average sum of variation (−37 copies), reflective of their numerous deletions. Similarly, the Low group tumors’ average sum of variation was 0.125 copies, close to the zero-value seen in the control samples; and the Amp group had an average sum of 82 copies. The significant difference in the average sums of variation (*p* < 0.0001) support there being 3 distinct CNV profiles for MCC rather than random distributions of deletions and amplifications in the tumors.

A similar trend between signature groups was seen when quantifying CNV on a per gene level with haploid deletions being more common than diploid deletions, and single copy amplifications being more common than multi copy amplifications (Figure 2C–F). Taken together, quantitative comparisons between the three structural variant signatures suggest that MCC tumors with genetic instability are dominated by either recurrent haploid deletions or recurrent amplifications in cancer associated genes.

### 2.3. VN-MCC Contains More Structural Variants Than VP-MCC

Comparing the average CNV per tumor for VP-MCC and VN-MCC samples we found that VN-MCC tumor samples contained significantly more structural variation per tumor (48.6) than VP-MCC samples (27.9) (Figure 2G). This is consistent with prior studies that also found higher rates of CNV in VN-MCC [21,25]. Interestingly, VP-MCC and VN-MCC showed no difference in the average sum of the variation, haploid deletions, diploid deletions, single copy amplifications, or multi copy amplifications (Figure 2H–L). Thus, although VN-MCC have more structural variants than VP-MCC on average, each virus status subtype contains similar frequencies of amplifications and deletions. The decreased average CNV count for VP-MCC was largely due to the fact that the eight tumors with the Low variant signature were exclusively VP-MCC. Accordingly, the structural variant signature of MCC tumors correlated with tumor virus status (two-tailed Fisher exact test, *p* < 0.005), with VP-MCC being more likely in the Low variant group and VN-MCC more likely in the deleted or amplified group. However, if a tumor was not in the Low variant group, the likelihood of having a deletion or amplification signature was independent of virus status (*p* = 1.0).

### 2.4. MCC Structural Variant Signatures Are Not Predictors of Survival

The three distinct CNV signatures observed in MCC tumors suggest differences in their biology that might impact disease progression. We used non-parametric Kaplan–Meier estimate to test for overall survival differences in patients based on the CNV signatures of their MCC tumors. Survival data was available for 29 of the 31 patients in the study. As shown in Figure S1, Kaplan–Meier survival estimates indicate that there is no statistical difference in survival between the three signature groups (*p* < 0.8857). Taken together, although the three signature groups reflect distinct patterns of genomic instability, any difference in survival was not detected in this cohort of patients.

## 3. Discussion

Merkel cell carcinoma generally arises on sun exposure skin, giving rise to the notion that UV mediated damage induces MCC [26,27,28,29]. UV-induced DNA damage is frequently seen in skin cancer and has been shown to cause genomic instability [30,31,32,33,34,35,36]. Oncoviruses also leads to genomic instability via virus integration or through the expression of viral oncogenes that alters the fidelity of replication [37]. Interestingly, although VP-MCC tumors do not have a significant enrichment of UV-induced sequence mutations, these tumors primarily occur on sun exposed regions of the skin and these tumors, like VN-MCC, also show genomic instability [21,25,38]. Here we used Nanostring Technologies nCounter system to examine the frequency of structural variation in 31 MCC tumors by quantifying amplifications and deletions in 86 cancer related genes. A number of the alterations found in our data are predicted to disrupt cell cycle regulation, including deletions of *RB1.* Deletions in the *RB1* locus or mutations that functionally inactive RB have been previously identified in MCC [11,13,15,21]. Loss of RB function is a well-established phenotype in a variety of cancers [39,40,41,42,43,44]. In VP-MCC the MCPyV large T antigen binds and inhibits RB, thereby releasing E2F to promote G1 to S phase transition through the cell cycle [45,46,47]. Interestingly, 5 (36%) of 14 VP-MCC also showed deletions in *RB1*, suggesting redundant inactivation of RB may play a role in either MCC onset or progression. Future studies to determine whether CNVs in *RB1* correlate with the presence or absence of specific sequence mutations may lead to a better understand of the pathophysiology of this disease.

Genomic amplifications can also dysregulate the cell cycle leading to tumorigenesis [23,48,49]. Our data shows numerous amplifications within the group 3 Amp cluster, some of which are well established proto-oncogenes known to be involved in the onset and progression of many different cancers. Specifically, we observed amplifications in *MYCL1* which was previously shown to me amplified in MCC [20,21,50]. Furthermore, the protein levels of MYC, which was also a gene loci amplified in the group 3 cluster, are stabilized in VP-MCC by the small T antigen binding and inhibiting the function of the F-box protein FBW7 [17]. In small cell lung cancer (SCLC), another neuroendocrine carcinoma, L-myc is thought to induce pre-rRNA synthesis and transcriptional pathways concomitant with ribosomal biogenesis [51]. A similar pathogenesis may be exploited in L-myc amplified MCC. Another interesting finding in our data is that tumors in the Amp signature cluster showed amplifications in *AKT3* whereas tumors with the Del signature had amplifications in *AKT2*, suggesting that both tumor types may utilize the AKT survival pathway for tumorigenesis. Inhibition of the AKT downstream target mTOR has already been implicated as a potential target for the treatment of MCC [52,53]. Moreover, gene mutations and amplification in AKT1 have been found in MCC through next generation sequencing studies [14,21]. Multiple lines of evidence suggestion that both L-myc and AKT could potentially be druggable targets in the treatment of MCC [14,50,51], and assessing CNV signature type may help predict which MCC tumors are more likely to respond to these treatments.

We observed a number of genomic structural variants previously unreported in MCC. Most notably, recurrent deletions of fragile histidine triad (*FHIT*) and recurrent amplifications in integrin β4 (*ITGβ4*). Interestingly, FHIT was shown to inhibit AKT activation leading to one mechanism by which FHIT decreases lung cancer cell survival [54,55]. Additionally, FHIT was shown to transcriptionally repress β-catenin [56], which is a downstream target of not only AKT but also of the WNT signaling pathway [57,58]. The deletions observed in *FHIT* could further implicate AKT in MCC. Intriguingly, ITGβ4 promotes metastasis through the induction of epithelial-mesenchymal transition in pancreatic ductal adenocarcinoma [59]. In addition, expression of ITGβ4, CD24 and Notch were shown to confer non-small cell lung carcinoma (NSCLC) propagation in clonogenic and othotoptic transplantation assays [60]. In MCC, amplification in *ITGβ4* might similarly promote proliferation and metastasis. Taken together, structural alterations in MCC tumors potentially alter a number of different pathways to increase tumor cells survival such as AKT, L-myc, RB, and β-catenin. Additionally, structural variation in *ITGβ4* could play a role in MCC metastases. Further work will be needed to test these potential associations.

The Nanostring technology used in this study allows for direct quantification of fragmented genomic DNA based on hybridization to barcoded probes for genes commonly amplified or deleted in cancers. The technology uses an average of 3 probes per gene, internal control probes to 54 invariant genomic regions, as well as spike-in process controls. Thus, copy number variants relative to similarly processed diploid control tissues can be reproducibly quantified from either FFPE or fresh-frozen tumor samples [61]. The heterogeneity of analyzing both FFPE and fresh-frozen samples from different institutions is a limitation of our study. It is noteworthy that the 8 tumors comprising the Low CNV cluster were all fresh-frozen samples from Memorial Sloan-Kettering, whereas the tumors in the Del and Amp clusters were FFPE samples. Although the fresh-frozen samples and controls met the same quality control endpoints as the FFPE samples and controls, it is possible that there were batch effects related to sample acquisition or fixation. Formalin fixation can cause DNA fragmentation, degradation, crosslinking, and adduct formation that can theoretically impact molecular studies [62]. In addition, unlike the FFPE controls, the fresh-frozen controls were normal adjacent tissues collected at the time of tumor excisions. Normal adjacent tissue has limitations as a control, but it is generally found to contain diploid DNA [63] and thus its use is unlikely to impact a pooled reference for CNV normalization. Despite these concerns, as discussed above, many of the recurrent CNVs observed in our study were previously reported in MCC tumors based on studies using other copy number assays [11,13,14,15,20,21,50], suggesting some accuracy in our data. Similarly, the observation that the average CNV load in VN-MCC is higher than the average for VP-MCC has also been reported in other studies [11,13,21]. Finally, our finding that individual VP-MCC tumors can have very few structural variants or can contain multiple amplifications or deletions is consistent with the results of Starrett et al., 2020 [21]. Nonetheless, the possibility that some artifact is contributing to the clustering of our data must be considered. Therefore, further studies will be needed to validate our findings and explore the functional implications of MCC tumors with different structural variant signatures.

## 4. Materials and Methods

### 4.1. Inclusion Criteria and Patient Samples

Archival cases of MCC were identified by a retrospective search for the diagnosis of Merkel cell carcinoma or neuroendocrine skin tumor in the Pathology Departments of the institutions. For cases where adequate tissue was available for analysis, the diagnosis was confirmed by an expert dermatopathologist (DK, KJB, or EYC) based on histopathology and immunostaining for diagnostic markers. After confirming a diagnosis of MCC and ensuring the sample was >75% tumor, tissue was cut for DNA extraction. For each case, available patient information was retrieved by clinical chart review. De-identified tissue samples and clinical data were sent to the NIH for analysis.

We performed CNV analysis on 23 formalin-Fixed Paraffin-Embedded (FFPE) MCC tumor samples and 8 fresh-frozen MCC tumor samples. FFPE tumors were collected from patients at the University of Pennsylvania and Marshfield clinic between August 1996 and April 2012. Fresh-frozen tumors and normal tissues were collected from patients at Memorial Sloan Kettering between July 1995 and August 2010. Control tissues used for normalization consisted of 2 FFPE normal spleen samples (controls for the FFPE tumors) and 3 fresh-frozen normal tissues adjacent to excised tumors (controls for the fresh-frozen tumors).

### 4.2. Genomic DNA Isolation

Genomic DNA (gDNA) was extracted from FFPE or fresh-frozen tumor with QIAamp DNA FFPE Tissue kit (Qiagen, Hilden, Germany) or the DNeasy Blood and Tissue kit (Qiagen, Hilden, Germany) respectively, according to the manufacturer protocols. Samples were treated with RNase A (Qiagen, Hilden, Germany) per manufacturer protocol. DNA concentration, 260/280 and 260/230 nm ratios were measured on a DeNovix DS-11 spectrophotometer (DeNovix Inc., Wilmington, DE, USA) prior to DNA fragmentation with Alu1 restriction endonuclease. Following Alu1 restriction digestion, fragmented DNA was analyzed on a 2100 Agilent Bioanalyzer (Agilent Technologies, Santa Clara, CA, USA). Representative electropherograms and gel images of Alu1 digested DNA from FFPE and fresh frozen samples can be found in Figure S2.

### 4.3. Virus Detection

Nested qPCR was used to detect the presence of the Merkel cell polyomavirus from gDNA. For step one, 20 ng of gDNA underwent 15 cycles of amplification with forward primer GGCAACATCCCTCTGATGAAAGC 3′ and reverse primer 5′ CCACCAGTCAAAACTTTCCCAAGTAGG 3′ using the KAPA2G Fast HotSStart PCR kit according to the manufacture protocol (Kapa Biosystems, Wilmington, MA, USA). Step two, 2 μL of step one product was amplified for 25 cycles in a OneStep Real-Time PCR System with forward primer 5′ CTTAAAGCATCACCCTGATAAAGG 3′ and reverse primer 5′ AAACCAAAGAATAAAGCACTGATAGCA 3′ using Power SYBR green master mix as per the manufacture protocol (ThermoFisher, Carlsbad, CA, USA). Primer set (forward 5′ CCACACTGCCCATCTCGGAGAC 3′ and reverse 5′ GCGGTGAGGTCCCTACGGCCTG 3′) for TPO was used as an endogenous control for quantitative PCR. gDNA from the VP-MCC cell line MKL1 and VN-MCC cell line UISO were used as controls to determine the presence or absences of the polyomavirus.

### 4.4. Cell Lines

UISO-MCC-1 [64] and MKL-1 [65] were previously described and grown in RPMI-1640 supplemented with 10% fetal bovine serum and 1% streptomycin/penicillin. Cell lines are sent out annually to be tested for authenticity via the Hum 16-Marker STR profile, interspecies contamination test and PCR evaluation for viruses and *Mycoplasma* which was performed by Idexx Bio Research.

### 4.5. Nanostring Prep and Run

A total of 600 ng of gDNA was process for Nanostring nCounter copy number variants as per the manufacturer protocol (Nanostring Technologies, Seattle, WA, USA).

### 4.6. Nanostring Data Analysis

Copy number for 86 genes for each tumor sample compared to the appropriate control samples were determined in nSolver according to manufacturer instructions. A heatmap of the normalized copy number data was generated in R using the gplots package and heatmap.2 code.

All statistical analyses were performed in GraphPad Prism (GraphPad Software, La Jolla, CA, USA). For each comparison, Grubbs’ Method was used to detect statistical outliers. For populations with normal distributions, *T*-test or one-way ANOVA were performed to assess differences between VP-MCC and VN-MCC or between clusters. For populations with statistical outliers, Mann–Whitney or Kruskal–Wallis test were performed to assess differences between VP-MCC and VN-MCC or between clusters. Significance was based on a *p*-value of less than 0.05.

## 5. Conclusions

We identified three distinct CNV signatures in MCC tumors. The observation that majority of MCC tumors demonstrate the Del structural signature, independent of virus status, suggests a shared pattern of genomic instability among most VP-MCC and VN-MCC tumors that promotes allelic deletions. In contrast, a subset of MCC tumors appear to be associated with mechanisms that promote genomic amplifications. A further subset of VP-MCC tumors are capable of progression with very few genomic structural alterations. As VP-MCC are known to have a very low somatic mutational burden, observing VP-MCC tumors with few CNVs suggests that viral oncogenes and epigenetic changes may be sufficient for tumorigenesis. Although the different CNV signatures were not associated with survival differences in MCC patients, the signatures were associated with recurrent changes in specific cancer pathways. It is possible that testing genomic structural signatures may help identify MCC patients more likely to respond to targeted therapeutic approaches.

## Figures and Tables

**Table 1:** Patient Summary.

MCC Sample	Sex	MCPyV Status	Age	Site of MCC	Specimen Code	Cluster	Tissue Source
MF1	Female	Negative	68	right upper arm	primary	Del	FFPE
MF2	Male	Negative	72	left hand	primary	Del	FFPE
MF3	Female	Negative	80	right gluteal	primary	Del	FFPE
MF4	Male	Negative	64	abdominal wall	primary	Del	FFPE
MF5	Male	Negative	89	left ala of nose	primary	Del	FFPE
MF6	Male	Negative	-	frontal scalp	primary	Amp	FFPE
MF7	Female	Negative	94	right scalp	primary	Del	FFPE
MF8	Female	Negative	-	lymph node	metastasis	Del	FFPE
MF9	Male	Positive	58	left thigh	primary	Del	FFPE
MF10	Male	Positive	67	left index finger	primary	Del	FFPE
MF11	Male	Negative	72	left cheek	primary	Del	FFPE
MF12	Male	Positive	-	right neck	primary	Del	FFPE
MF13	Female	Negative	-	right leg	primary	Del	FFPE
MF14	Female	Negative	100	right forehead	primary	Del	FFPE
MF15	Male	Negative	93	left cheek, nose	primary	Amp	FFPE
MF16	Female	Negative	74	left buttock	primary	Del	FFPE
MF18	Female	Negative	-	right forearm	primary	Del	FFPE
MF19	Male	Negative	77	right face	metastasis	Del	FFPE
MF20	Male	Positive	75	top of head	primary	Del	FFPE
MF21	Male	Negative	87	right wrist	primary	Amp	FFPE
MF22	Female	Negative	88	forehead	primary	Amp	FFPE
MF23	Male	Positive	81	left cheek	primary	Del	FFPE
UP1	Female	Positive	75	left brow	-	Amp	FFPE
MSK1	Female	Positive	80	lymph nodes	metastasis	Low	Frozen
MSK2	Male	Positive	73	pancreas	metastasis	Low	Frozen
MSK6	Male	Positive	53	groin	metastasis	Low	Frozen
MSK13	Female	Positive	62	skin	primary	Low	Frozen
MSK19	Male	Positive	59	skin	primary	Low	Frozen
MSK20	Female	Positive	63	skin	primary	Low	Frozen
MSK21	Male	Positive	87	skin	primary	Low	Frozen
MSK24	Male	Positive	82	lymph nodes	metastasis	Low	Frozen

## Data Availability

Data is contained within the article or supplementary material.

## Supplementary Materials

The following are available online at https://www.mdpi.com/2072-6694/13/5/1134/s1, Figure S1: The three genomic structural variant clusters are not predictors of overall survival, Figure S2: Bioanalyzer analysis of Alu1 cut DNA from FFPE and fresh frozen MCC tumor samples, Table S1: Normalized genomic copy number at 86 gene loci for MCC tumor samples.
