# Supplementary material for: Distinct Signatures of Genomic Copy Number Variants Define Subgroups of Merkel Cell Carcinoma Tumors
Source: Cancers (Basel). 2021 Mar 6;13(5):1134. doi: 10.3390/cancers13051134 (PMC7961454; doi:10.3390/cancers13051134)
Supplement: Supplementary file 1 [file cancers-13-01134-s001.pdf]

Supplementary Materials

Distinct Signatures of Genomic Copy Number Variants Define Subgroups of Merkel Cell Carcinoma Tumors

Natasha T. Hill, David Kim, Klaus J. Busam, Emily Y. Chu, Clayton Green and Isaac Brownell

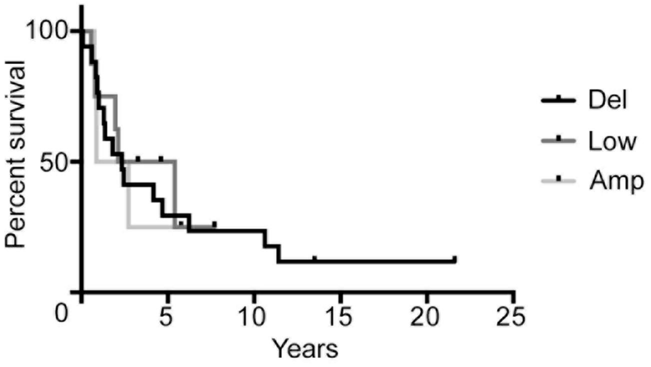

Figure S1. The three genomic structural variant clusters are not predictors of overall survival. Overall survival comparing the three clusters as determined by Kaplan-Meier survival analysis.

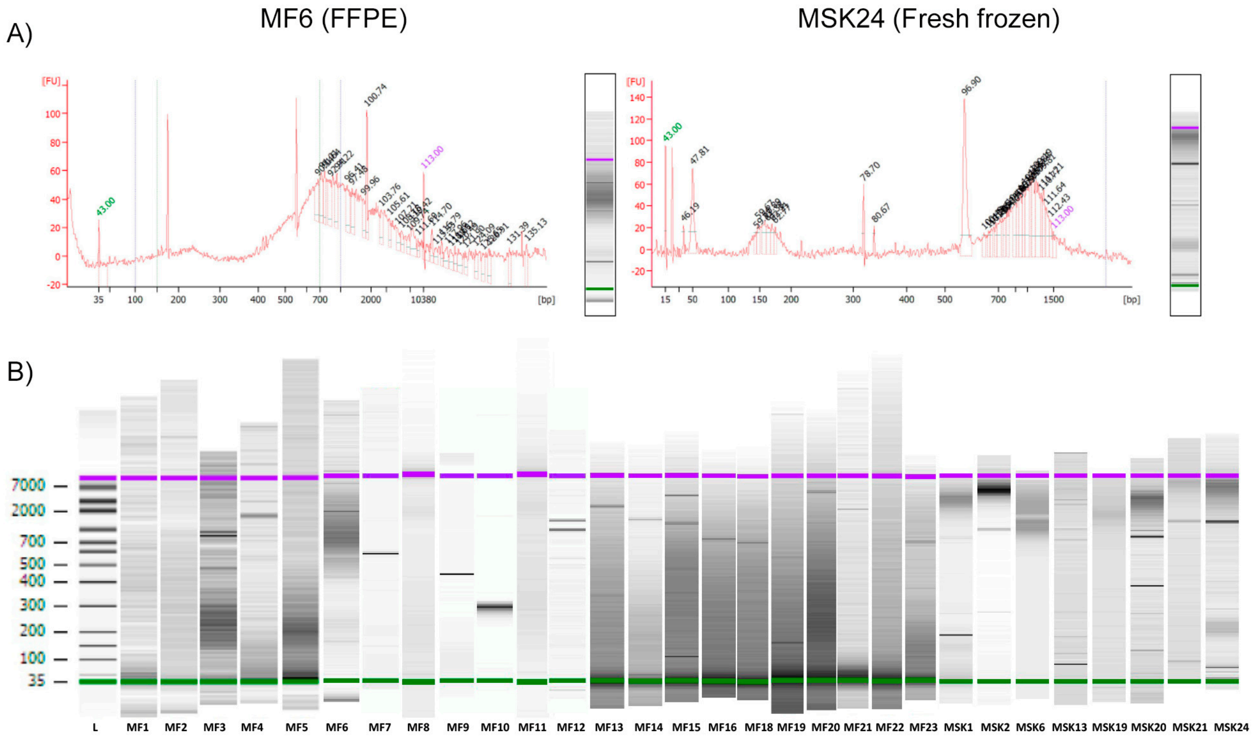

Figure S2. Bioanalyzer analysis of Alu1 cut DNA from FFPE and fresh frozen MCC tumor samples. As per the Nanostring protocol, extracted FFPE and fresh frozen tumor DNA was fragmented by digestion with Alu1 restriction endonuclease for 2 hours at 37 °C. Following digestion, tumor DNA was subjected to bioanalyzer analysis. (**A**) Representative examples of a FFPE and fresh frozen bioanalyzer trace following Alu1 digestion. (**B**) Gel image collage of bioanalyzer analysis of the Marshfield (MF, FFPE) and Memorial Sloan Kettering (MSK, fresh frozen) tumors following Alu1 digestion. L, DNA ladder.

Table S1. Normalized genomic copy number at 86 gene loci for MCC tumor samples.

Gene Name	MF1 (VP)	MF2 (VN)	MF3 (VN)	MF4 (VN)	MF5 (VN)	MF6 (VN)	MF7 (VN)	MF8 (VN)	MF9 (VN)	MF10 (VN)	MF11 (VN)	MF12 (VN)	MF13 (VN)	MF14 (VN)	MF15 (VN)	MF16 (VP)	MF18 (VP)	MF19 (VP)	MF20 (VN)	MF21 (VN)	MF22 (VN)	MF23 (VP)	UP1 (VP)	MSK01 (VP)	MSK02 (VP)	MSK06 (VP)	MSK13 (VP)	MSK19 (VP)	MSK20 (VP)	MSK21 (VP)	MSK24 (VP)
MAGI3	1.4	1.4	1.7	1.4	0.7	3.5	1.3	1.8	2	1.6	1.4	1.5	2.1	1.2	5.9	1.9	1.7	1.7	2	2.3	2.5	1.7	3.1	2.1	2	1.6	2.3	2.1	2	3.3	2
REG4	1	0.3	1.4	1.2	0.4	3.1	0.9	1.2	1.5	0.8	1.3	1.2	1.6	0.8	6	1.3	1.4	1.2	1.4	2.7	3	1.5	4	2.1	1.8	1.6	2.2	2	1.8	2.9	1.8
MCL1	1.7	2.1	2.3	2	2.4	2.7	1.8	3.2	2.3	1.8	2.2	1.9	2.3	2	5.2	1.8	1.7	1.9	1.7	3.6	5.5	1.8	6.5	2.2	2.1	1.9	2	2.7	1.8	2.7	2.3
MDM4	1.4	1.3	1.4	1.3	1.5	3.1	1.1	1.6	1.7	1.2	1.5	1.6	1.6	1.3	4.8	1.3	1.4	1.4	1.2	3.5	5.3	1.5	4.7	1.8	1.9	1.6	1.6	2.6	1.7	3.3	4.2
AKT3	1.5	1.2	1.9	1.6	1.8	3.2	1.6	1.7	1.8	1.2	1.7	1.6	1.8	2.3	4.8	1.5	1.7	1.6	1.6	3.8	5.9	1.5	4	2.2	1.8	2.2	2.2	2	1.8	2.8	1.7
TP73	1	2.5	2.3	2	3	1.8	2	2.7	2.3	1.8	3.6	2.1	2.4	2.4	3.4	2.3	2.2	1.7	1.8	1.6	3.4	2.1	1.3	2.3	2	1	2.1	1.9	1.9	2	1.4
MYCL1	1.2	0.4	1.7	1.3	1.7	2.7	1.1	1.6	1.5	1	2.5	1.2	1.8	1.4	4.6	1.3	1.5	1.2	1.4	2.4	4.4	1.5	4.1	2	1.8	1.5	1.8	1.8	1.6	2.5	2.7
CDKN2C	1.5	1	1.8	1	1.6	3.3	1.2	1.2	2.1	1.1	2.7	1.5	1.9	1.8	6.5	1.5	1.4	1.3	1.4	2.9	8.8	1.3	7.1	1.9	1.6	1.5	1.8	1.9	1.6	2.6	3
JUN	1.3	1.4	1.7	1.3	2.1	2.4	1.3	2	1.8	1	2.6	1.4	2	1.8	5.1	1.8	1.8	1.7	2	2	4.2	2.1	2.1	1.9	1.7	1.6	2	1.9	1.7	2.8	2.4
MYCN	1.6	1.9	2.6	1.7	1.5	2.7	1.2	2	2.1	1.9	1.8	1.6	2.2	2.2	2.9	1.8	1.9	1.6	2.1	2.2	2.4	1.7	3.9	2.2	2	1.8	2.1	2	1.8	2	2.1
REL	1.3	1	1.3	1.3	1	2.8	0.9	1.1	1.8	1.1	1.6	1.6	1.5	1.5	2.7	1.1	1.5	1.4	1.3	2.7	3.1	1.2	5.1	2.3	2	1.4	2.1	2.1	1.7	2.1	2.6
PRKCI	1.6	2.7	1.6	1.4	1.8	3	1.8	2.5	1.7	1.6	2.1	1.7	1.9	1.6	2.7	1.5	1.6	1.6	1.5	2.8	4.3	1.5	3.7	2.3	2	1.5	2.2	2	1.8	1.8	2.1
PIK3CA	1.2	1.6	1.4	1	1.6	2.4	1.3	1.7	1.5	1.6	1.7	1.6	1.4	1.5	2.3	1.2	1.4	1.5	1.2	2	3.5	1.3	2.8	1.6	1.7	1.2	1.8	2.1	1.7	2.4	4.5
DCUN1D1	1.2	1.4	1.4	1.3	1.6	3.2	1.5	2	1.8	1.3	1.6	1.6	1.6	1.5	2.9	1.3	1.5	1.5	1.4	2.7	4.5	1.5	4.1	2	2.2	1.6	2.1	2.2	2	2.1	2.4
FHIT	1.5	0.4	1.9	1.4	0.7	1.6	1.1	1.7	1.8	1.4	2	1.8	1.8	2	2.4	1.6	1.8	1.4	1.8	2.1	1.5	1.6	3.1	2.2	1.9	1.6	2.3	2	1.9	2	1.8
MITF	1	0.6	1.4	1.2	0.5	2.6	0.9	0.8	1.9	0.9	1.6	1.3	1.7	1.5	3.5	1.5	1.6	1.3	1.5	3.5	2.2	1.5	5.6	1.9	1.8	1.3	2	1.9	1.8	2	1.7
PDGFRA	0.8	0.8	1.1	1	0.8	1.8	1	0.9	1.5	0.8	1.4	1.2	1.3	1.3	2.6	1.1	1.2	1.1	1.2	2.5	2.3	1.2	5.5	1.8	1.9	1.3	1.7	1.8	1.7	1	1.4
KIT	1.2	1.2	1.7	1.6	1.2	2.4	1.6	1.6	2.3	1.2	1.8	1.4	1.8	2.1	3.5	1.5	1.6	1.7	1.3	3.4	3.7	1.5	6.3	2.2	2	1.5	2.2	2.2	2	1.2	1.4
KDR	1.2	0.9	1.5	1.7	1.1	2.2	1.3	1.5	1.9	1.2	1.7	1.4	1.7	1.7	3.2	1.4	1.6	1.7	1.3	3.2	3.1	1.5	4.6	2.2	2	1.6	2.4	2.1	2	1.2	1.5
APC	1.1	0.6	0.9	1.1	0.9	3.5	0.8	0.9	1.7	0.7	1.5	1.3	1.6	0.9	3.1	1.3	1.6	1.2	1.6	2.5	2.9	1.6	3.6	2.3	2	1.7	1.3	2	1.9	2	1.8
TERT	1.4	1.9	3.6	1.4	1.7	1.7	2	1.9	1.7	1.4	1.6	1.6	2.1	3	1.8	2	2	2	2	1.3	2.1	2.1	1.2	2	1.9	1.8	3.4	1.8	2.4	2.7	2
SKP2	0.9	1.1	2.2	1.5	0.9	3.4	1.3	0.9	1.7	1	1.6	1.4	1.7	1.9	3.1	1.1	1.6	1.4	1.1	3.6	7.1	1.3	6.2	1.9	1.8	1.5	3.5	2	2.3	2	1.7
PDE4D	1.5	0.8	1.9	1	0.9	3.6	1	1.2	2	1.1	1.5	1.5	1.7	1.6	3.3	1.5	1.6	1.8	1.8	3.4	4	1.5	5.1	2.1	2	1.4	2.2	2.1	2.1	2.3	1.8
MYB	1.7	1.1	1.4	1.6	1.2	1.5	1.5	1.3	1.4	1.6	1.6	1.6	1.5	1.3	2.2	1.3	1.5	1.7	1.3	2	3.2	1.2	3.1	1.9	2	1.7	1.9	2	2.5	2.2	2.5
MAP3K5	1.5	0.8	1.6	1	0.8	2.3	0.9	1.8	1.8	1.2	1.2	1.5	1.5	1.6	2.9	1.8	1.6	1.7	1.1	3.7	5.3	1.7	5	2.5	2.4	2.6	2.7	2.4	3	2.4	2.6
PARK2	1.3	0.6	1.6	1.1	0.8	2.4	1.2	0.5	1.7	1.8	1.4	1.3	1.7	1.9	3.5	1.6	1.7	1.4	1.4	3.2	5.1	1.7	4.2	2.3	2	2.3	2.5	2.1	2.7	2.1	2.2
E2F3	1.3	0.8	1.6	1.3	1	4.1	1.2	1.2	1.6	1.8	1.3	1.4	1.4	1.3	5.2	1.6	1.6	1.4	1.5	2.4	3.8	1.4	3.2	2.2	1.9	2.7	2.2	2.2	2.5	1.9	2.3
CDKN1A	1.9	1.7	2.1	1.9	1.6	2.2	1.8	1.9	1.9	3.6	1.9	1.7	2	1.7	3.4	1.9	1.7	2	1.5	1.9	2.8	1.8	2.5	2	1.8	2.5	1.9	2.1	2.2	1.8	2.4
VEGFA	2.1	2.3	1.9	1.8	1.6	2.6	2.2	1.8	2	3.6	2	2	2.1	2.1	3.9	2.2	1.9	2.3	1.9	2.3	3	2.1	3.3	1.9	1.7	2.7	1.8	1.9	2.3	1.7	2.3
MET	1.2	1	1.5	1.3	1.2	2.5	0.9	1.2	1.6	1.5	1.6	1.7	1.9	1.5	3.5	1.5	1.6	1.4	1.6	2.6	4.3	1.3	4.4	2.1	2.1	1.4	2.1	2.2	2	2.2	2
SHH	2	1.6	2.4	2.2	2.3	1.8	1.8	2.2	2.5	1.9	2	2.1	2.5	2.6	2.8	2.3	2.4	2.8	2.4	1.4	2.6	2.9	1.9	2.1	2.1	1.6	2.2	2	1.9	1.9	2.1
EGFR	1.6	1.1	1.9	1.7	1.3	2.7	1.7	2	1.9	1.4	1.9	1.7	1.8	2.1	3.6	1.6	1.7	1.7	1.7	2.6	3.6	1.8	3.1	2	1.8	1.5	2.1	1.8	1.7	1.8	1.6
CDK6	1.2	1.5	1.8	1.5	1.1	3.7	1.5	2.1	1.5	1.3	1.6	1.6	1.7	2.1	4.1	1.4	1.6	1.4	1.8	2.7	4.6	1.5	2.8	2.1	1.4	1.6	2.3	2.1	2	2.2	1.8
YWHAZ	1	0.7	1.2	1	0.8	5	1	0.9	1.7	0.8	1.3	1.3	1.4	1.3	3.5	1.1	1.5	1.3	1	3.2	3.7	1.3	4.7	3.1	2.1	1.4	2.2	2.1	1.9	3.1	2.5
MYC	1.7	2.2	1.8	1.7	1.7	3.6	1.9	1.8	2.3	1.7	1.8	1.9	2	1.7	2.5	1.9	1.8	1.9	2.1	2.4	2.6	1.9	4.1	2.9	1.9	1.7	2.1	1.8	1.6	0.9	2.3
CSMD1	0.7	0.4	1.1	0.9	0.7	4	0.8	0.9	1.6	0.5	1.2	1.1	1.3	1.5	4	1.2	1.3	0.9	1.2	3.1	3.1	1.1	4.4	1.2	1.9	1.4	2.1	1.9	1.8	1	2.1
WHSC1L1	1.3	0.9	1.5	1.6	1.1	2.5	1.4	1.5	1.9	1	1.8	1.5	1.7	1.2	2.3	1.3	1.5	1.4	1.2	2.7	2.9	1.6	3.8	1.2	2	1.5	2	2	1.8	0.9	2.3
FGFR1	1.2	0.8	1.4	1.4	1.2	2	1.5	1.4	1.8	1.3	1.8	1.6	1.6	1.6	1.9	1.4	1.6	1.6	1.4	2.4	2.1	1.8	2.7	1.3	2.2	1.6	2.1	2.1	2	1	2.7
C8orf4	1.4	1	1.8	1.8	1.5	3.5	2.2	1.9	2.4	1.7	2.2	1.9	1.8	1.8	2.9	1.5	1.7	1.9	1.4	3.1	3	1.7	3.4	1.5	2.5	1.8	2.7	2.5	2.4	1.3	2.6
TRAF2	1.5	2.5	1.7	1.3	1.4	2.3	1.4	2	1.9	1.1	2.3	1.8	1.7	1.9	1.5	1.8	1.9	1.9	1.6	2	1.6	2.1	1.8	2.5	2.2	2.6	2.3	2.1	1.9	1.9	3.3
CDKN2A	1.2	0.5	1.6	1.6	1.1	4.2	0.9	1.2	2.3	0.8	1.6	1.2	1.9	0.7	2.9	1.5	1.6	1.7	1.4	2.1	2.5	1.5	3.8	1.5	2	1.4	2	2.1	2	2.2	1.8
MELK	0.9	0.3	1	0.8	0.7	2.5	0.5	0.6	1.3	0.5	1.2	1.1	1.4	0.8	2.2	1.3	1.5	1.1	1.3	2.3	2.3	1.4	2	2.4	2.2	2.5	2.4	2.4	1.8	2.1	2.2
PTPRD	1	0.6	1.2	1.1	0.9	4.9	0.8	1.2	1.4	0.8	1.3	1.2	1.4	0.7	3.3	1.2	1.4	1.2	1.1	2.9	2.9	1.2	4.2	1.9	1.9	1.4	2.2	1.9	1.8	1.9	1.2
PTEN	1.3	1.1	1.8	1.4	1.3	2.1	1.1	1	1.9	1.1	1.9	1.5	1.6	1.6	0.8	0.8	1.5	0.9	1.3	3.2	3.4	1.5	4.9	2.2	2	1.4	2.2	1.6	2	2.2	1
YAP1	1.3	0.9	1	1.1	1	2.7	0.8	1.4	1.7	1.1	1.4	1.6	1.4	1.7	2.4	1.3	1.7	1.6	1.3	2.8	2.9	1.5	2.6	2.1	1.9	1.5	2.2	1.9	2.4	2	1.3
BIRC2	1.6	0.9	1.2	0.9	1.1	2.5	0.8	0.6	1.8	1.6	1.5	1.7	1.6	1.5	2.1	1.4	1.6	1.6	1.5	2.7	2.9	1.7	3.2	2.3	2.3	1.5	2.3	2.4	2.8	2.3	1.6
WT1	0.8	0.3	0.9	0.7	0.7	1.9	0.5	0.5	1.2	0.7	0.6	0.9	1.2	1.2	2.5	1	1.3	1.2	0.9	2.6	2.7	1.3	3.4	2.1	2	1.6	2.2	2	2.5	2	1.6
CCND1	1.1	1.1	1.7	1.5	1	2.1	0.8	0.6	1.7	1.4	1.4	1.5	1.9	1.7	1.9	1.7	1.8	1.8	1.4	1.6	1.4	2.1	1.8	2	1.7	1.6	1.9	1.7	2.3	1.6	1.4
ORAOV1	1.4	1.2	1.2	1	0.8	2.7	0.9	1.6	1.4	1	1.3	1.4	1.4	1.7	2.3	1.4	1.6	1.6	1.3	2.6	2.6	1.8	3.4	2.1	2.1	1.6	2.1	2	2.5	1.8	1.5
FADD	1.2	1.5	1.7	1.5	1.2	2.3	1.1	1.2	1.8	1.3	1.5	1.8	1.6	1.5	2.3	1.5	1.5	1.5	1.4	2.1	2.1	1.7	3.9	1.9	1.8	1.4	1.8	1.7	2.2	1.7	1.3
GAB2	1.2	1.2	1.5	1.2	1.1	2.7	0.8	1.1	1.4	1.1	1.4	1.3	1.6	1.7	2.5	1.4	1.6	1.4	1.5	2.2	2.5	1.4	3.3	2.4	2.2	1.7	2.4	2.3	2.7	1.9	1.7
KRAS	2.3	2.3	1.9	1.8	1.5	2.2	2.1	1.9	2.3	1.9	2.4	2.2	1.9	1.8	1.9	1.5	1.9	2.4	1.5	2.7	3.1	1.7	3.7	2.1	1.9	2	2.1	2.1	1.8	2	1.7
CCND2	1.2	1.8	1.6	1.2	1	2.3	1.5	2	1.6	1.4	1.4	1.7	1.6	1.7	2.2	1.4	1.5	1.4	1.4	2.1	2.3	1.5	3.4	1.8	1.8	2	2	1.9	1.8	1.9	1.3
CDK4	1.3	1.1	1.2	1.4	1.1	2.1	1	1	1.5	1.6	1.4	1.3	1.5	1.5	2	1.3	1.5	1.6	1.4	1.9	2	1.4	3	2	1.8	2.1	1.9	1.9	1.8	1.9	1.7
HMGA2	1.4	1.7	1.8	1.7	1.1	3.1	1.5	1.7	2	1.7	1.8	1.8	1.8	1.8	2.9	1.5	1.5	1.7	1.7	3.2	3.6	1.4	5.3	2.1	1.8	2.1	2.2	2	1.8	2	1.5
DYRK2	1.3	1.4	1.7	1.4	1.1	3.4	1.3	1.2	2.2	0.9	1.3	1.6	1.8	1.6	2.8	2	1.6	2.1	1.7	2.8	3.1	1.7	4.8	2.2	2	2.3	2.3	2.2	2	2.2	1.5
MDM2	1.5	0.7	1.4	1.3	1.1	2.8	1.1	1.9	1.6	1	1.7	1.6	1.4	1.2	2.6	1.4	1.5	1.3	1.3	3	3.2	1.5	3.8	2.2	2	2	2	2	1.8	2	1.6
IRS2	1.4	1.9	2.2	1.8	2.2	4.5	2.3	2.4	1.9	2.4	1.2	2	2.2	1.4	2	2.1	2.1	2.2	2.5	2.2	2.7	2.1	3.2	2.2	2.1	2.9	2.6	2.4	2.1	2.2	3.2
BRCA2	2.1	1.3	2	1.8	2.3	2.5	1.1	2.6	2	2	1.3	2.3	2.1	1.1	1.7	2.1	2.1	2.1	2	2.4	2.8	1.9	1.7	2	1.7	1.9	2	2.2	1.8	2.4	3.2
FOXO1	1.3	1	1.3	1.4	0.7	2.9	1	0.8	1.6	1.4	1	1.5	1.7	1.2	1.9	1.3	1.5	1.2	1.2	2.6	3.1	1.5	2.6	2	1.7	2	2.1	1.9	1.7	1	1.8
RB1	1.1	0.5	1	0.7	0.4	2.5	0.5	0.6	1.4	0.9	0.7	1.1	1.5	0.8	1.9	1.3	1.5	1.3	1.2	2.5	2.6	1.7	2.1	2	1.8	2.2	2.1	2.1	1.8	1.1	1.7
GPC5	1.3	0.9	1.5	1.3	1.3	5.1	1.5	2	1.9	1.3	0.9	1.6	1.7	1	2.1	1.4	1.6	1.3	1.5	2.9	3.7	1.4	4.7	1.9	1.8	2	2.2	1.9	1.8	2	1.7
BCL2L2	3.7	2.5	2.1	1.8	2.1	3	1.6	1.3	2.1	2.4	1.7	1.9	1.9	1.7	2.5	1.9	1.8	2.1	1.7	2.5	2.5	1.8	3.3	1.9	1.8	1.8	1.6	1.8	1.7	1.7	2.8
NKX2-1	1.9	2.6	2.4	2.2	3.1	1.6	2.3	3.1	2.7	2.2	2.4	2.5	2.6	2.6	1.6	2.7	2.5	2.7	2.4	1.8	1.5	3	1.1	2	1.8	2.4	2.1	1.8	1.8	1.9	2.8
NKX2-8	2.1	3.6	2.3	2	2.6	1.8	2.1	3	2.9	2.6	2.5	2.1	2.9	2.5	1.9	2.5	2.5	3	2.2	1.7	1.4	2.6	1.5	1.9	1.7	2.2	2	1.9	1.6	1.9	3
PAX9	0.9	0.8	1.4	1.2	1.4	4	0.9	1	1.7	0.9	1.7	1.2	1.6	1.2	3.4	1.3	1.2	1.2	1.1	2.4	2.6	1.4	3.9	1.9	1.9	2	2	2	1.9	2.1	2.4
IGF1R	1	1.1	1.3	1.1	0.8	3.1	1	0.8	1.6	0.8	1.5	1.2	1.5	1.5	2.8	1.3	1.6	1.2	1.2	2.6	2.5	1.4	3.8	2.2	2	2.2	2.2	2	1.9	1	1
MAP2K4	0.9	1.1	1.6	1.2	1.1	3.9	0.9	1.9	1.6	1.1	1.3	1.4	1.7	1.7	3.5	1.4	1.5	1.3	1.3	2.9	1.9	1.3	4.8	2.2	1.9	1.6	2.4	1.5	1.9	1.1	1.3
MAPK7	1.3	2.7	1.5	1.6	1.5	2.1	1.1	1.6	1.7	3.1	2.2	1.7	2.1	1.7	2.3	1.8	1.5	1.6	1.3	2.3	1.4	1.7	4	2.2	1.9	1.6	2	1.4	1.8	1	2
NF1	0.8	0.7	1	1	0.7	3.4	0.6	0.8	1.6	1.2	1.2	1.1	1.4	0.7	3.1	1.1	1.3	1.1	0.9	2.8	4	1.5	4.5	2.2	2.1	1.6	2.3	2.1	1.9	2.1	2
ERBB2	2.4	3.4	2.7	2.4	1.9	1.9	1.7	3.4	2.5	2.3	2.4	2.3	2.5	2.6	2	2.4	2.2	2.7	1.8	2.2	2	2.2	2.2	2.2	2	1.5	2	1.8	1.8	1.8	2.2
BRCA1	1.4	1.7	1.3	1.3	1.4	2.6	1	1.4	2	2	2.1	1.8	1.8	1.4	2.3	1.3	1.6	1.9	1.2	2.5	3.1	1.6	3.2	2.3	2.1	1.6	2.1	2.1	1.8	1.8	2.2
RPS6KB1	1.3	0.9	1.1	1	1.1	1.8	0.7	0.3	1.6	0.9	1.4	1.4	1.6	1.8	1.8	1.2	1.5	1.5	1.2	1.7	1.7	1.4	2.4	2.5	2.3	1.7	2.3	2.4	2.5	2	3.4
GRB2	1.4	1.1	1.7	1.5	1.3	2.7	1	1.5	1.9	1.7	1.9	1.7	1.6	2.6	2.2	1.6	1.8	1.8	1.6	2.8	2.5	2	2.7	2.7	2.5	1.9	2.5	2.3	2.8	2.1	2.2
ITGB4	2.5	2.4	2.4	2.6	2.2	1.6	2	3.5	3.2	2.2	3.4	2.8	2.6	4	1.6	2.9	3	3.4	2.3	2.3	1.8	2.9	1.6	2.6	2.4	1.8	2.4	2.1	2.8	2.1	2.5
TP53	1.5	1.7	2	1.9	1.5	1.5	1.3	2.3	1.8	1.4	2	1.8	1.9	1.8	1.3	1.5	1.9	1.7	1.7	1.8	0.9	1.9	1.8	2.3	2.2	1.7	2.3	1.5	1.8	1.1	2.2
DCC	1.3	0.9	1.4	1	0.9	3.5	1.4	0.9	1.9	1	1.6	1.3	1.4	1.2	3	1.2	1.3	1.1	1.5	2.9	3.9	1.2	4.3	2.1	1.8	1.5	3.1	2.1	1.9	2.2	1.9
CCNE1	1.4	1.4	1.3	1.2	1.6	2.4	2.1	1.3	1.9	1.4	1.7	1.6	1.5	1.6	2.4	1.4	1.7	1.8	1.3	3.2	3.4	1.7	5.5	2.1	1.9	2.1	1.1	1.8	2.3	1.7	1.9
AKT2	2.2	2.1	2	2.5	2.5	1.9	2.7	3	2.4	2.5	2.8	2.8	2.4	2.6	1.7	2.1	2.4	2.8	1.7	2.6	2.4	2.3	2	2.4	2.1	2.5	2.4	2	1.9	1.9	1.4
BBC3	1.9	2.2	2	1.9	2.4	1.5	1.7	2.3	1.8	2	1.8	1.9	2	1.9	1.5	2.1	1.9	1.9	2.2	0.8	1.3	2.2	1.6	2.4	2.1	2.3	2.1	1.8	1.7	1.7	1.5
BCL2L1	1.6	1.3	1.6	1.9	1.1	2.9	1.2	1.3	2.1	1.2	1.8	1.7	1.6	1.5	2.8	1.5	1.7	1.6	1.5	2.9	2.9	1.7	3.9	2.2	2.1	2.1	2	2.7	1.8	3	2.3
NCOA3	1	0.5	0.9	0.7	0.8	2.9	0.8	0.8	1.2	1.3	1.1	1.1	1.3	1.3	2.3	1.2	1.3	1.3	1.1	2	2.3	1.4	2.9	2.2	2.1	2.3	2.2	2.2	2	2.3	2.4
ZNF217	1.5	1.5	1.9	1.7	1.5	2.9	1.9	1.9	1.8	2.1	2	2	1.9	1.9	2.4	1.8	1.7	1.9	1.7	2.6	2.7	2	3.2	2	1.9	2	2	1.9	1.7	2	1.8
AURKA	0.9	0.5	1.1	0.9	0.9	3.1	1	1	1.2	1	1.1	1.1	1.2	0.8	3	0.9	1.1	0.9	0.9	2.7	2.6	1.1	4.4	2	1.9	1.8	1.8	1.9	1.7	1.9	1.9
EEF1A2	2.1	1.5	1.9	1.8	2.3	1.6	2.9	1.9	2.9	2.3	2.4	2.4	2.5	2.2	1.5	2.5	2.6	2.6	2.2	2.1	1.5	3	1.6	2.2	1.9	2.3	1.8	1.8	1.8	1.7	2
CRKL	1.2	1.1	1.4	1.3	0.9	2.9	0.8	0.9	1.6	1.2	1.4	1.3	1.5	1.6	2.5	1.3	1.5	1.3	1.6	2.2	2.4	1.5	3.4	2.2	2.2	1.4	2	1.9	1.7	1.8	2

Copy number was determine using the nSolver 3.0 software. VN; VN-MCC, VP; VP-MCC, MF; Marshfield Clinic, UP; University of Pennsylvania, and MSK; Memorial Sloan Kettering.
